# Addressing Individual Values to Impact Prudent Antimicrobial Prescribing in Animal Agriculture

**DOI:** 10.3389/fvets.2020.00297

**Published:** 2020-05-28

**Authors:** Laurel E. Redding, Cecilia Brooks, Christine B. Georgakakos, Greg Habing, Leah Rosenkrantz, Michael Dahlstrom, Paul J. Plummer

**Affiliations:** ^1^School of Veterinary Medicine, University of Pennsylvania Kennett Square, PA, United States; ^2^Department of Psychology and Family Studies, Mississippi University for Women, Columbus, MS, United States; ^3^Biological and Environmental Engineering, Cornell University, Ithaca, NY, United States; ^4^Department of Veterinary Preventive Medicine, The Ohio State University, Columbus, OH, United States; ^5^Department of Geography, Simon Fraser University, Burnaby, BC, Canada; ^6^Greenlee School of Journalism and Communication, Iowa State University, Ames, IA, United States; ^7^Veterinary Diagnostic and Production Animal Medicine, Iowa State University, Ames, IA, United States; ^8^National Institute of Antimicrobial Resistance Research and Education, Ames, IA, United States

**Keywords:** antimicrobial, social science, communication, values & beliefs, animal agriculture

## Abstract

Antimicrobial resistance is a growing public health threat driven by antimicrobial use—both judicious and injudicious—in people and animals. In animal agriculture, antimicrobials are used to treat, control, and prevent disease in herds of animals. While such use generally occurs under the broad supervision of a veterinarian, individual animals are often treated by farm owners or managers. The decision to administer antimicrobials is therefore influenced not only by the clinical situation but also by the motivations and priorities of different individual actors. Many studies have examined the drivers of external forces such as costs, workload and time constraints, or social pressures on antimicrobial use by veterinarians and producers, but none have explored the role of individually held values in influencing decision-making related to antimicrobial use. Values are deeply held normative orientations that guide the formation of attitudes and behaviors across multiple contexts. Values have been shown to be strongly tied to perceptions of and attitudes toward polarizing topics such as climate change, and preliminary evidence suggests that values are also associated with attitudes to antimicrobial resistance and stewardship. In this article, we draw on lessons learned in other fields (human health care, climate change science) to explore how values could be tied to the extrinsic and intrinsic factors that drive antimicrobial use and prescribing in animal agriculture. We also provide suggestions for ways to build a bridge between the veterinary and social sciences and incorporate values into future research aimed at promoting antimicrobial stewardship in animal agriculture.

## Introduction

Antimicrobial resistance is a growing public health crisis, driven, in part, by the widespread use of antimicrobials in both people and animals (1). Addressing this crisis within animal agriculture must account for multiple levels of decision-making by prescribers (e.g., veterinarians) and users (e.g., farmers), considering the influencers of those decisions at each level. Considering both the prescriber and the user as distinct agents for change is critical, as the prescriber is seldom present when the end-user administers an antimicrobial treatment (2, 3). In such cases, the decisions regarding antimicrobial use are less influenced by expert veterinary knowledge and more significantly influenced by other factors such as policy, guiding principles of practice, or social pressure (including social capital) (4). Changing the behavior of an antimicrobial prescriber or user is a complicated and difficult task that requires more than the passive transfer of knowledge. Knowledge alone is not sufficient to decrease use or improve antimicrobial stewardship (5, 6). Accordingly, antimicrobial stewardship efforts must focus on addressing the beliefs, perceptions, and values held by these agents to most effectively influence behavioral change in antimicrobial prescription and usage.

This article aims to build a bridge between the veterinary and social sciences by exploring the role of individual values in the decision-making process around antimicrobial prescribing and use. This article evaluates insights gained from values-based research in other healthcare and social examples, and considers how these values-based inferences intersect with sociologic impacts on social and cultural capital. Connecting these bodies of knowledge will help us to better achieve our antimicrobial stewardship objectives.

## Antimicrobial use in Animal Agriculture: Current Practices and Motivators

In animal production systems, antimicrobials are used for disease treatment, control, prevention, and, in parts of the world, growth promotion. While global best practices for treating individual diseases among veterinarians and animal producers have not been instituted, guidelines of judicious use have been put forth and include, among others, preventing disease occurrence through improved livestock management systems or vaccination, reducing unnecessary antimicrobial use by assuring appropriate drug selection and dosing, assuring veterinary oversight of antimicrobial use in animals, and restricting the use of antimicrobials for growth promotion purposes (7, 8). While the evidence for these recommendations is admittedly low-quality (8), it does suggest that reducing overall antimicrobial usage in animal agriculture results in modestly decreased antimicrobial resistance in animals and arguably people (9, 10). Since all uses of antimicrobials, including prudent use, impart selective pressure for the emergence of resistant organisms, a major focus of research in veterinary medicine, public health, and policy involves finding ways to improve antimicrobial stewardship and use in animal agriculture.

Most of the knowledge related to interventions targeting antimicrobial prescribing behavior is derived from human medicine. Antimicrobial prescribing by physicians has been shown to be strongly influenced by behavioral and cultural determinants (11, 12), and a systematic review of antimicrobial stewardship interventions in human medicine suggested that persuasive measures (e.g., aimed at achieving voluntary behavior change) generally resulted in more sustained changes than restrictive measures (5). More recent work has argued that there is a critical need in human medicine to reframe the antimicrobial stewardship efforts based on the underlying values that drive prescribing behavior (13). While there is overlap between how human and veterinary medicine is practiced, animal agriculture represents a fundamentally different antimicrobial prescribing ecosystem, where population medicine and economics play a more significant role in treatment and management decisions. Interventions designed to improve antimicrobial stewardship in animal agriculture are further complicated by the fact that, while veterinarians provide oversight of antimicrobial prescribing, individual treatment decisions are often made by farm owners or employees following standard operating procedures outlined by the veterinarian. For example, in the United States, farm personnel may be involved in identifying individual animals or groups of animals that require antimicrobials based on predetermined guidelines for antimicrobial use established by the veterinarian. While veterinary oversight is broadly present, the majority of individual treatments are often initiated without consulting a veterinarian about that specific case (2, 3) and by different actors (e.g., farm owner, employee) who may be influenced in that decision process by varying motivations and priorities, such as the cost of diagnostic tests, prior experience, and risk avoidance treatments (14, 15).

From a social science perspective, studies have examined antimicrobial users' and prescribers' perceptions of antimicrobial resistance and antimicrobial stewardship. As might be expected, producers, and veterinarians appear to hold a wide range of beliefs and perceptions on antimicrobial use and resistance in food animal production, with views often differing both across study contexts and within regions where farming practices are comparable (16–21). Respondents most often report external driving forces on their prescribing/using behavior, including economic factors (3, 15, 22–24), workload and time pressures (18, 21, 25), social pressures such as perceived expectations of other parties (e.g., clients, patients, product purchasers, and other farmers) (18, 25–31), and previous experiences (15, 22). Personal factors such as desire for recognition, fear of shame among peers, and the intrinsic satisfaction of doing a good job (32, 33) have also been found to influence decision-making among farmers. These concepts are perhaps best embodied in the sociologic concept of the “good farmer,” whereby farmers also value non-economic rewards or “capital” in decision making. Founded on Bourdieu's theory of capital (34, 35), “good farmers” are not only identified by economic capital (market sales and other mercantile transactions) but also strive for social capital (perception of social networks and ability to meet mutual obligations) and cultural capital (measured by perceived prestige derived through certification programs and symbolic measures such as how the livestock appear) (36–38). At a practical level this cultural capital of the “good farmer” is derived from the everyday practices and skills of the farmer, but most importantly is measured by external observation of peers. Herein, lies the concept of gaining cultural capital by having the tidiest farmstead, straightest planting rows and healthiest, biggest livestock— all of which are measures that can be easily assessed by knowledgeable peers observing the operation (36, 38). Even though the measures might not have positive economic gain (and in some cases, such as spending more money on keeping the property tidy, might actually decrease net revenue), farmers consider these intrinsic motivators in their decision making (36–38). These intrinsic motivators often stem directly from an individual's underlying value set. While a significant body of research has addressed extrinsic and some intrinsic factors in relation to antimicrobial prescribing and use (17, 23, 29, 39), far fewer studies have examined the deeper underlying values of these factors, especially with regards to their influence on how knowledge related to antimicrobial resistance and use is interpreted and applied. This knowledge gap is critical to address if we are to truly understand and change human behaviors that contribute to antimicrobial resistance in animal agriculture.

## What are Values?

Values have been defined in a number of ways in the psychosocial literature, but in essence, they are conceptions of desirable end states that reflect what is important to us in our lives (40, 41). They transcend specific situations, inform the selection, or evaluation of behavior and events, and guide the formation of attitudes and behaviors across multiple contexts (41). Values are thought to be cognitive representations of the biological, social interactional, and social institutional needs of an individual (41) and are thus inherently socially driven. Thus, the “good farmer” is driven by what he or she perceives as a desirable end state within the context of the social interactions and social institutions that govern his or her life.

Attitudes and behaviors about scientific topics come from applying scientific knowledge in service of an underlying value system (42). In short, science may describe and explain the world, but it can never tell society what ought to be done. Thus, for many controversial scientific issues such as antimicrobial resistance, climate change, or vaccinology, additional scientific knowledge does not always lead to a greater consensus. In fact, the individuals most knowledgeable about the science are also often the most polarized (42), as the same scientific knowledge is being applied to serve different values. Knowing what values an audience will likely use to interpret a message offers the communicator an avenue to align the message around something that already matters to the audience. Therefore, identifying the values of the antimicrobial end-user is relevant in judicious use and implementation of antimicrobials on farm operations.

## What can we Learn From Other Examples?

While few studies have explored the role of normative values in the decision-making of antimicrobial prescribers and users in animal agriculture, a body of literature has examined this topic in relation to other topics such as human health care and climate change. Insights from this literature can be derived to better understand the interactions between values, perceptions, and decision-making of farmers and veterinarians related to antimicrobial use.

### Values in Health Care

While improving antimicrobial stewardship in human medicine might be thought of as a natural parallel to improving antimicrobial stewardship in animal agriculture, there are many differences that exist between the two—the most obvious being in the different values we hold for human life vs. animal life. While all veterinarians and farmers likely place animal welfare high on the list when treating with antimicrobials, culling economically inefficient animals is common, and occasionally entire flocks or herds may be depopulated if disease is rampant (43). Obviously, these decisions would be unimaginable for a physician. Consequently, while physicians and veterinarians both want to ensure the best health for their patients, it may be challenging to apply the insights learned from values-based research related to antimicrobial stewardship in human medicine to animal agriculture. Nonetheless, values-based research in human medicine can offer important insights to antimicrobial stewardship in general. For example, when examining the relationship between values and antimicrobial prescribing behaviors, physicians have reported being intrinsically motivated to deliver care that is grounded in the best available science and the ethics of medicine (4). However, social views or changes in policy and regulations can drive change in prescribing behaviors. For example, regulatory changes in prescribing standards may bring about attitudinal changes among physicians resulting in a need to re-examine their intrinsic motivations used in their decision-making process (4). A recent exploration of the role of values in human antimicrobial stewardship efforts identified temporal short-sightedness, individualization, marketization, and human exceptionalism as key value drivers hindering progress in human medicine (13). One proposed solution was to encourage a more solidaristic model, where responsibility for outcomes related to antimicrobial use are shared by both the individual and the broader institutional hierarchy and translated into new legal, administrative, and bureaucratic norms (13). While one might suspect that these findings could apply to veterinarians given their similarities in roles and responsibilities to physicians, such research has yet to be conducted. In contrast, some studies have demonstrated that health related government policy changes and regulatory oversight is sometimes interpreted negatively by farmers as poorly informed and not consistent with “good farmer” practices (44). In such cases, new animal health policies may be poorly adopted.

### Values in Climate Change

Antimicrobial resistance in animal agriculture represents a societal type of risk, where the impacts are distant and diffuse rather than immediate to the individual making the decision. A related societal risk where research into the underlying social factors is more developed is climate change. An individual's attitude toward climate change depends on a number of values-based factors, including social risk perception, social trust, and religiosity. According to cultural theory, risk perception is a social construction that is strongly influenced by how an individual feels society should be organized across two dimensions (45). The “group” dimension ranges from individualism to communitarianism and conveys how strongly an individual feels bonded to a social group. The “grid” dimension ranges from hierarchy to equalitarianism and conveys the amount of social control and structure people desire in their social group (45). Where a person falls on these scales was found to be significantly associated with their views on climate change (42). In general, people with communitarian worldviews were more likely to accept that climate change exists than people with individualistic worldviews (42, 46), and people with egalitarian worldviews tended to be more accepting than people with hierarchical worldviews (47, 48). Both of these axes of sociality are highly correlated with political affiliation (49), and a stark divide over the perception of climate change along political ideology lines has been thoroughly demonstrated (47, 50–52). Specifically, it has been posited that people with individualistic worldviews resent restrictions on individual choices, especially with regards to decisions that could affect economics and commerce, while people with hierarchical worldviews place more value in rules and regulations from those higher up in the hierarchy.

Other factors that tend to be associated with the axes of sociality and perceptions of climate change are social trust and religiosity. People with a general distrust of social institutions and with high levels of religiosity tend to express skepticism about climate change, and vice-versa (51, 53–55). While religious beliefs are said to compete with science over “moral, epistemological, and ontological issues” (54), social trust supplements knowledge and reduces the complexity of a situation or decision-making process (56). In the face of a lack of knowledge, people turn to trusted sources for guidance on decision-making.

At this point, while there is relative consensus that values influence attitudes toward climate change, very little formal testing of different climate change education and communication strategies tailored to individual values has been performed. In an experimental study, Kahan et al. found that nearly identical newspaper articles titled and describing a solution to global warming as either “anti-pollution” or “nuclear” produced different effects on audience depending on where they fell along the grid-group dimensions (57), thus demonstrating how values impact an individual's perception of and actions related to a situation. In another study, investigators sought to use the constructive power of social norms (i.e., community mindedness) to successfully reduce energy consumption among consumers by providing them with data on energy consumption of their peers (58). However, in the absence of experimental evidence for tailored communication strategies in communicating climate change messaging, researchers have proposed ways in which such messaging could theoretically be effective (59, 60). For example, to decrease climate change skepticism in an ideologically conservative audience, Zia and Todd recommend re-framing the issue of climate change as either (1) a security issue by emphasizing the risks and impacts of drastic climate change, or 2) as a religious issue causing “pain and suffering for fellow humans, animals and plants” (61). Brownlee et al. suggest that in an audience skeptical of science and institutions (i.e., with decreased social trust), educational content should avoid charts, graphs, and references to science in favor of personal stories (62). In all cases, there appears to be a consensus that tailoring should focus on intrinsic values (such as civic duty) rather than extrinsic values (such as economic factors) (62).

### Expected Impact of Values on Antimicrobial Stewardship in Animal Agriculture

Scientific literature is beginning to explore the role of social trust and values within the context of antimicrobial stewardship in animal agriculture. Several studies have assessed the relationship between social trust and attitudes toward antimicrobial use/prescribing among veterinarians or producers. These studies suggest that the variation in social trust influences perception of antimicrobial use and resistance (17, 63, 64). For example, personal experience has been shown to be a strong driver of antimicrobial use/prescribing and other health-related decision-making among some veterinarians and producers, even superseding antimicrobial use guidelines and regulations developed by experts and authority figures (65, 66). Anecdotally, individuals with limited trust in social institutions expressed the belief that the regulatory agencies developing recommendations and regulations are ill-informed about the realities faced by producers and more interested in restricting their behavior than promoting the public good (66). Additionally, while very little information is available on the association between religiosity and perceptions of antimicrobial use and resistance in the veterinary literature, one study found that farmers who identified as Amish and Mennonite generally used antimicrobials less frequently than other farmers (54). However, it is unclear whether the less frequent use of antimicrobials was specifically related to their cultural or religious background or to their preference for a different type of farming (e.g., small scale, low inputs, low outputs) that results in less disease and therefore less need for antimicrobials.

The scientific literature from other contexts allows prediction of how underlying values may relate to antimicrobial stewardship. Audiences with hierarchical worldviews would likely support the pathway to judicious antimicrobial use through increased regulation coming from authoritative experts. Audiences with individualist worldviews would likely view these same regulations as heavy handed or out-of-touch, and instead place more value on the situational knowledge of an individual operation. Audiences with egalitarian worldviews would instead likely focus on the similarities across situations and how everyone involved could do a little bit toward the larger goal. These differences in perspective have been discussed with regards to human medicine in antimicrobial stewardship (13). Differences in deeply-held values may suggest that, for instance, introducing a solidaristic model toward antimicrobial stewardship will be very difficult in the face of individualistic values. None of the views are incompatible with each other, but misaligning the message with the value would greatly diminish its impact. Qualitative studies soliciting veterinarians' and producers' attitudes toward antimicrobial use regulation provide preliminary evidence of such attitudes and perceptions: for example, cattle producers expressing negative sentiments toward the Veterinary Feed Directive specifically described it as top-down “over-reach” by the government (67). In contrast, farmers that expressed strong desires to be perceived by their peers as “good farmers” (i.e., communitarianism worldview) were more likely to endorse measures to promote judicious antimicrobial use (29, 43).

It seems likely that the intersection of cultural theory and the “good farmer” construct (38) further impacts antimicrobial stewardship and decision making by farmers. For instance, the cultural capital and social capital perceived by an individual farmer may be unique to the worldview that aligns with their values. These differences might be manifest in different cultural capital measures based on underlying values, with some farmers most interested in being a “good farmer” by minimizing antibiotic use and others most motivated to be perceived as being a “good farmer” by having the largest, heaviest livestock or the most rapid rate of weight gain.

## How do we Improve our Communication in Light of These Issues?

Because an individual's values are thought to be critical to their perceptions of and attitudes toward complex phenomena such as antimicrobial resistance, future initiatives addressing these complex issues should connect the veterinary science of antimicrobial stewardship with the social science of decision making (68, 69). Every act of communication should be considered as a two-way social negotiation. In the context of climate change, for example, Ballantyne suggests that communication on this topic must be a constitutive process of producing and reproducing shared meanings, requiring a shift “to a perspective where all participants—senders and receivers— become coauthors or co-creators of meaning and where cultural and social contexts are recognized as important influential factors” (57).

What does this look like if we take a similar approach when attempting to address antimicrobial resistance? First, we call on researchers to listen to various actors in the chain of decisions leading to livestock antimicrobial exposure and explore their underlying values that drive how they interpret veterinary knowledge and act on those perceptions. High quality literature has documented attitudes held by these various stakeholders (18, 20, 21, 30, 70) but little is known on the values these actors hold. Moreover, as we have explored above, it is clear that what is known becomes fuzzier under the lens of context: antimicrobial type, farm type, and other contextual factors may alter the values held by these actors in unexpected ways. It is also necessary to develop a better understanding of the drivers of cultural and social capital (34–36) for different subsets of farmers (based on cultural theory) in order to develop effective communication campaigns. These gaps in knowledge are major roadblocks to effecting positive change with regards to the more prudent use of antimicrobials in livestock.

We also call on decision-makers to understand that attitudes and values are equally, if not more, important than the external factors of knowledge and awareness for behavioral change. One should be skeptical anytime someone claims that more information or knowledge by itself will solve the problem. Likewise, one should push back anytime someone disparages beliefs or emotions surrounding the topic of antimicrobial resistance as being unimportant. Finally, lest policy makers despair that immutable values are what ultimately dictate an individual's approach to antimicrobial use and that change is therefore unlikely to happen, one can be encouraged in observing that external influences such as education, regulation, or social pressures do appear able to change prescribers' fundamental attitudes toward antimicrobial stewardship. For example, differences in attitudes toward antimicrobial stewardship among veterinary practitioners with differing numbers of years in practice point to the ability of education and contemporaneous factors to influence values related to antimicrobial prescribing (71). Similarly, a social pressure campaign and resultant regulatory changes related to antimicrobial dispensing and prescribing in France was anecdotally able to change veterinarians' perceived responsibility for antimicrobial resistance and influence them to adjust prescribing habits (72).

In terms of concrete changes in research and policy to address this gap in knowledge, different approaches can be used. For example, evaluation of the success of antimicrobial stewardship policies should include collection of both numerical data on metrics of success as well as nuanced qualitative data to understand how individual factors such as values influence the implementation of the policy. Clack et al. (73) provide an example of how this can be done: in evaluating the effectiveness of two evidence-based healthcare associated infection reduction strategies in intensive care units across 14 hospitals in 11 European countries, these authors conducted in-depth interviews with various hospital staff and performed observations of practices prior to and 1 year after the intervention. They were able to identify how sociocultural factors (i.e., related to values) specific to each hospital influenced the success of the interventions and thus provide insight into how to improve adoption of policy measures.

There is also a need for stewardship interventions that tailor the language and delivery method of interventions to the values of the intended audience. As we discussed previously, this is already the recommended approach in climate change science communication (60–62). Additionally, such recommendations have been made in the context of human medicine (74), where it was observed that the norms and values of a specific medical specialty (e.g., collectivism in the internal medicine service vs. individualism in the surgery service) impacted decisions and outcomes related to antimicrobial stewardship (75, 76). For example, stewardship recommendations could be promoted to communitarianism-minded individuals by highlighting their potential impact on the community. Alternatively, they could be rolled out and advocated for by an authority figure to a hierarchical-minded audience.

Truly understanding the values of an audience will allow the communicator to position the relevant knowledge to allow the audience to support their values more fully. As antimicrobial resistance continues to grow worldwide, shifting our mindset, and connect the veterinary science of antimicrobial stewardship with the social science of decision making will be of utmost importance to optimizing antimicrobial stewardship efforts in animal agriculture and assure the continued utility of our limited antimicrobial resources.

## Author Contributions

All authors contributed to the conception and design of the manuscript, wrote sections of the manuscript, contributed to manuscript revision, and read and approved the submitted version.

## Conflict of Interest

The authors declare that the research was conducted in the absence of any commercial or financial relationships that could be construed as a potential conflict of interest.
